# Editorial: Sound Perception and the Well-Being of Vulnerable Groups

**DOI:** 10.3389/fpsyg.2022.836946

**Published:** 2022-02-10

**Authors:** Qi Meng, Pyoung Jik Lee, Hui Ma

**Affiliations:** ^1^Key Laboratory of Cold Region Urban and Rural Human Settlement Environment Science and Technology, School of Architecture, Harbin Institute of Technology, Ministry of Industry and Information Technology, Harbin, China; ^2^School of Architecture, University of Liverpool, Liverpool, United Kingdom; ^3^School of Architecture, Tianjin University, Tianjin, China

**Keywords:** sound perception, soundscape, vulnerable groups, health, restoration, theoretical framework, methodological tool

## Introduction

Sound environment is a significant factor to be considered in building sustainable and healthy urban communities and cities (Kang and Schulte-Fortkamp, 2018). The International Organization for Standardization (ISO) defines soundscape as an acoustic environment that is perceived or experienced and/or understood by a person or people in context (ISO, 2014). In general, the current soundscape researches have mainly focused on urban spaces (e.g., parks, open spaces, and streets) and functional buildings (e.g., hospitals, schools, offices and residences). The experience of environment can result in either positive or negative perceptual outcomes, which are in turn related to people's well-being and emotion state. In terms of a positive soundscape, for instance, natural environment as well as music can induce positive emotions and help restoring attention and reducing mental fatigue. On the other hand, as the World Health Organization (WHO) Environmental Noise Guidelines report (WHO, 2018), noise is a considerable public health issue which would lead to hearing loss, annoyance, sleep disturbance, and even cardiovascular and metabolic disease.

In most cases, the related experiments have discussed individual differences on soundscape evaluations such as comfort, tranquility, capacity for restoration and soundscape quality according to people's different demographic and social characteristics (such as age, gender, and income level), as well as their behavior status (such as sitting, resting and strolling). However, the differences between groups are not often given enough prominence in environmental psychology studies, particularly research on vulnerable groups.

Vulnerable groups involve human samples considered particularly susceptible to stress or undue influence in a research setting. Existing researches show that the elderly have special challenges for hearing in noise; noise would disturb patients' sleep and the noise level should not exceed 30 dBA at night inside the hospital; noise would even disturb children's cognitive development; and people with high noise sensitivity are more susceptible to noise (Xie et al., 2009; Zeng et al., 2021). Besides, a Review of Evidence in the WHO European Region which analyzed articles published between 2010 and 2017 (Dreger et al., 2019) indicated that there was a trend of higher environmental noise exposures in groups with lower socioeconomic position. However, there are still so many questions in sound perception of vulnerable groups, such as, “What kind of people are the vulnerable groups in urban or building sound environments?,” “What are the differences between vulnerable groups and general persons in sound perception?,” and “What sound environment is the best choice for different vulnerable groups?”

Therefore, this special issue aims at gathering articles talking about soundscape perception of vulnerable groups (including but not being limited to the aged, children, patients, and the low-income). The focus included theoretical aspects (e.g., relationships between sounds and psychological, physiological as well as behavioral pattern aspects, for instance, human health, psychological/physiological restoration, and emotion changes) and methodological aspects (e.g., protocols and procedures to gather acoustic and psychological data).

## Research Themes

Considering the broad scope of the call for papers, the topics and research questions addressed by the submissions we received were very diverse. Looking retrospectively at them, we tried to identify common themes and eventually clustered them under three main categories. There were: (1) adverse effects caused by noise on vulnerable groups, (2) potential benefits brought by soundscape for vulnerable groups, and (3) restoration of the sound environment in patients. The experience of the environment can result in either positive or negative perceptual outcomes, which are in turn related to well-being and emotional state on vulnerable groups. Thus, contributions to this particular research strand were particularly welcome to advance the scientific conversation on these issues.

### Adverse Effects Caused by Noise on Vulnerable Groups

Benito et al. described the relationship between the traffic noise level and the perceived annoyance in the inhabitants of a city on the Northern Border of Mexico. The results show that the population is desensitized to traffic noise and does not perceive it as an annoyance. The flow of vehicles and the type of vehicles are the significant factors for the propagation and increase in the traffic noise levels. Women present a considerable appreciation of traffic noise perception instead of younger people who demonstrate a higher tolerance to high-level exposure. Yang, Feng et al. conducted a 7-month noise level (*L*_Aeq_) measurement on a construction site of a reinforced concrete structure high-rise residential building in northern China. It was done to explore the acoustic environment on the construction site, the environmental experience of construction workers, the impact of noise on hearing and on-site communications, and the corresponding influencing factors. Qu and Tsuchiya investigated the relationship between wind turbine noise (WTN), noise perception, and self-reported health of people, and controlled for background characteristics of the residents in urbanized areas. The dose-response relationship was found between WTN and annoyance, moderated by age and degree of education. Cai et al. investigated the acute physiological effect of different noise-sensitive groups by indoor-level noise stimulus experiments under laboratory conditions, by observing heart rate variability (HRV) indicators, including Standard Deviation of NN intervals (SDNN), Low Frequency/High Frequency (LF/HF), and Heart Rate (HR).

### Potential Benefits Brought by Soundscape for Vulnerable Groups

Yang, Wang et al. focused on the usually neglected acoustic environment and its effect on drivers' physiological state and driving behaviors. The results indicated that different sound scenarios in the highway tunnel showed significant differences in vehicle speed and steering wheel angle. Zhu et al. compared through soundscape evaluation including an analysis of the dominance of various sound sources, noise annoyance, and the perceptual dimensions of soundscape. Jiang et al. proposed a three-dimensional pleasure arousal dominance (PAD) emotional model to indicate whether the perception of one's music environment has influences on college students' emotion during communication in different indoor conditions including spatial function, visual and sound atmospheres, and interior furnishings. Zhang Zhang et al. conducted an independent sample non-parametric test to determine the significance of the differences between environmental evaluation results for each evaluation dimension and to summarize the compositions of sound and space elements in the positive and negative influence spaces. Liu, Xu et al. explored the reality of the soundscape preferences of Chinese urban residents in the general public landscape in the post-pandemic area, and then proposed design recommendations to meet the practical needs of people s preferences for landscape especially soundscapes in the post-pandemic area. Zhang, Kong et al. distributed questionnaires to believers and tourists inside and outside several well-known Han Chinese Buddhist temples in China to analyze the relationship between evaluations of temple soundscapes (including the overall acoustic environment and preferences for typical sounds) and mental health and the role of religious belief-related factors in this relationship.

### Restoration of Sound Environment in Patients

Cui et al. used the methods of field observation, sound measurement, and questionnaire survey to explore the sound perception and preference of the elderly in the main indoor public space of a nursing home in Harbin. The results revealed that in terms of the temporal and spatial distribution of sound pressure level (SPL), the unit living space had the highest SPL, which was above 60 dBA. The reverberation time (RT) of the unit living space, medical and health care center corridor, was 2.15 and 2.13 s, respectively. Liu, Wang et al. used facial expression recognition software (FaceReader) to explore the influence of different sound interventions on the emotions of older people with dementia. The field experiment was carried out in the public activity space of an older adult care facility. The results showed that, in the music intervention, the valence in the first 80 s helps to predict dominance and acoustic comfort; in the stream sound intervention, the first 40 s helps to predict pleasure and acoustic comfort; for the birdsong intervention, the first 20 s helps to predict dominance and arousal.

## Concluding Remarks

While the three themes discussed above certainly do not cover the full range of questions being discussed in the soundscape studies on vulnerable groups, they do detect a few “hot topics” and areas of interest for researchers and practitioners in the field. The psychological theory underpinning the environmental sounds perception processes could still be considered (at least) as evolving. There is a clear interest in making a connection with health and well-being frameworks and also an outlook toward the design and co-creation of open public spaces. Virtual reality techniques are now commonly used in perceptual experiments, together with onsite surveys and the analysis of “big data” from public sources. Going forward, it will be essential to include all possible stakeholders in the debate: the public, researchers, practitioners, artists, and professionals with different skills and expertise. This will help generate and test new hypotheses and triangulate methodologies and results.

## Author Contributions

All authors listed have made a substantial, direct, and intellectual contribution to the work and approved it for publication.

## Funding

This work was supported by the National Natural Science Foundation of China (NSFC) (Grant Number 51878210).

## Conflict of Interest

The authors declare that the research was conducted in the absence of any commercial or financial relationships that could be construed as a potential conflict of interest.

## Publisher's Note

All claims expressed in this article are solely those of the authors and do not necessarily represent those of their affiliated organizations, or those of the publisher, the editors and the reviewers. Any product that may be evaluated in this article, or claim that may be made by its manufacturer, is not guaranteed or endorsed by the publisher.
